# Gastrointestinal failure in intensive care: a retrospective clinical study in three different intensive care units in Germany and Estonia

**DOI:** 10.1186/1471-230X-6-19

**Published:** 2006-06-22

**Authors:** Annika Reintam, Pille Parm, Uwe Redlich, Liina-Mai Tooding, Joel Starkopf, Friedrich Köhler, Claudia Spies, Hartmut Kern

**Affiliations:** 1 General Intensive Care Unit, Tartu University Clinics, Tartu, Estonia; 2 Department of Anaesthesiology and Intensive Care, University of Tartu, Tartu, Estonia; 3 Department of Anaesthesiology and Intensive Care, DRK Kliniken Berlin, Berlin, Germany; 4 Department of Anaesthesiology and Intensive Care, Charité Universitätsmedizin Berlin, Berlin, Germany

## Abstract

**Background:**

While gastrointestinal problems are common in ICU patients with multiple organ failure, gastrointestinal failure has not been given the consideration other organ systems receive. The aim of this study was to evaluate the incidence of gastrointestinal failure (GIF), to identify its risk factors, and to determine its association with ICU mortality.

**Methods:**

A retrospective analysis of adult patients (n = 2588) admitted to three different ICUs (two ICUs at the university hospital Charité-Universitätsmedizin Berlin, Germany and one at Tartu University Clinics, Estonia) during the year 2002 was performed.

Data recorded in a computerized database were used in Berlin. In Tartu, the data documented in the patients' charts was retrospectively transferred into a similar database. GIF was defined as documented gastrointestinal problems (food intolerance, gastrointestinal haemorrhage, and/or ileus) in the patient data at any period of their ICU stay. ICU mortality, length of stay, and duration of mechanical ventilation were assessed as outcome parameters.

**Results:**

GIF was identified in 252 patients (9.7% of all patients). Only 20% of GIF patients were identifiable at admission. GIF was related to significantly higher mortality (43.7% vs. 5.3% in patients without GIF), as well as prolonged length of ICU stay (10 vs. 2 days) and mechanical ventilation (8 vs. 1 day), p < 0.001, respectively. Patients' profile (emergency surgical or medical), APACHE II and SOFA scores and the use of catecholamines at admission were identified as independent risk factors for the development of GIF. Development of GIF during ICU stay was an independent predictor for death.

**Conclusion:**

Gastrointestinal failure represents a relevant clinical problem accompanied by an increased mortality, longer ICU stay and mechanical ventilation.

## Background

Patients with multiple organ failure (MOF) are the most challenging group of patients in modern intensive care. The gastrointestinal system is one of the clinically important systems in MOF, with strong evidence of bacterial translocation in ICU patients supporting the concept of the gut being a motor of MOF [1; 2]. On one hand, it has been suggested that nosocomial infection caused by enteric bacteria is directly related to the development of MOF in some patients. On the other hand, this may be a secondary phenomenon and a symptom rather than a cause of MOF [3]. The functional status of the gastrointestinal tract has not been studied in full depth and consensus criteria for diagnosis of gastrointestinal failure (GIF) have not been established. Some authors describe GIF as "gastroparesis and intestinal ileus" [4], while others define it as gastrointestinal haemorrhage [5].

None of the scoring systems for organ dysfunction and severity of illness widely used today take the function of the gastrointestinal tract into account. The multiple organ failure (MOF) score, one of the first attempts to quantify the severity of organ dysfunction and failure, originally included gastrointestinal tract when it was developed in 1985 [6]. Revision of the score 15 years later however, concluded that GIF should not be considered in the assessment of the MOF score due to problems in definition and reliability [7]. The SOFA score is another well-established score for assessing organ dysfunction or organ failure over time, and has proven useful for evaluating morbidity and predicting ICU mortality [8,9]. The six organ systems used in calculating this score do not include GIF. It has not been proven whether an assessment of the gastrointestinal system would add predictive power to SOFA estimations of ICU survival. However, recent studies have shown that intraabdominal hypertension on admission is associated with severe organ dysfunction during the ICU stay and the development of intraabdominal hypertension during the ICU stay is an independent outcome predictor [10].

Despite a large number of human studies suggesting that the gastrointestinal tract contributes significantly to morbidity and mortality in critically ill ICU patients, data on risk factors, time-course, prognostic importance, and suggestions for clinical evaluation of gastrointestinal function are surprisingly poor and inconsistent in existing literature. The present study was undertaken to identify the incidence of and independent risk factors for GIF in ICU patients and to investigate its impact on ICU mortality.

## Methods

A retrospective evaluation of the data of all adult patients admitted to three different ICUs (two 11-bed ICUs at the Charité – University Medicine Berlin, Germany and one 10-bed ICU at Tartu University Hospital, Estonia) during the year 2002 was performed.

In Berlin, the ICUs had a primarily cardiosurgical profile. The Estonian ICU was a general ICU treating medical and surgical emergencies, but not cardiosurgical or neurosurgical patients. Data recorded in a computerized database were used for the study in Berlin. In Tartu, the data documented in the patients' charts was retrospectively transferred into a similar database. We analysed 47 variables from first ICU day, including patients' profile (elective surgical, emergency surgical or medial), age, sex, diagnose group, source of admission, ICU, APACHE II, SOFA, SAPS II, mean arterial pressure, heart rate, Glasgow coma scale, pH, CVP, PEEP, operation time, reoperation, bleeding, haemoglobin, haematocrit, WBC, platelets, glucose, C-reactive protein, albumin, protein, bilirubin, creatinine, urea, pO_2_/FiO_2_, lactate, antithrombin III, readmission, reintubation, mechanical ventilation, use of catecholamines, use of blood products, use of thrombolytics, sedation, enteral nutrition, renal replacement therapy, low cardiac output syndrome, renal failure, liver failure, septic shock, diabetes, reanimation.

Severity of illness was assessed using the Acute Physiology and Chronic Health Evaluation (APACHE II)score [11] at admission and Sequential Organ Failure Assessment (SOFA) [8]score daily.

### Group assignment

GIF was defined as the presence of at least one of the following gastrointestinal problems documented in patient data during their ICU stay: food intolerance, gastrointestinal haemorrhage, and ileus. Food intolerance was defined as the inability to feed the patient via nasogastric tube due to vomiting or nasogastric aspirate volumes larger than those previously given enterally. Gastrointestinal haemorrhage was defined as visual presence of blood in nasogastric tube aspirates or in stool. Ileus was defined as intestinal obstruction due to inhibition of bowel motility. Prophylactic antacids and prokinetic drugs (metoclopramide) were routinely used in all units.

Ethical approval was obtained at the Charité – University Medicine Berlin and ethical aspects of the study were consulted with the ethicist of the University of Tartu. Written informed consent was considered not necessary for the study, as it is a retrospective analysis of our usual everyday work. No special interventions were used. All the data were impersonalised within the respective units before analysis, and no harm could be weighed against benefit.

### Statistics

SPSS (Version 11.5 SPSS Inc., Chicago, IL) software was used for statistical analysis.

Univariate analysis for comparison of GIF vs. nonGIF patients was performed using Mann-Whitney U-test for continuous variables and chi square test for categorical variables. Analysis of variance (ANOVA) was used for comparison of groups according to patients' profile.

To identify the risk factors for the development of GIF univariate analyses of parameters recorded on ICU day 1 were performed using independent samples t-test or Mann-Whitney U-test for continuous parameters. Chi square tests were used for categorical variables. Parameters with p < 0.01 were considered as (univariately) highly predictive parameters for GIF and were entered into the multiple logistic regression model. Multiple logistic regression analysis was performed to identify independent risk factors for GIF. Stepwise regression was used to ascertain the most important parameters. Stepwise multiple logistic regression was also applied to test the severity scores and GIF as independent risk factors for death.

## Results

### 1. Patients' characteristics

2588 patients were analysed during the study period. Majority of them (1771 patients) were postoperatively admitted elective cardiosurgical patients. Among 581 patients hospitalised due to surgical emergency, 310 were cardiac cases and 271 patients with surgical infections (pancreatitis, peritonitis, soft tissue infections, etc.) or traumatic injuries. No isolated neurosurgical patients were treated during the study period. Among 236 medical patients 94 patients were resuscitated from cardiac arrest. Other main reasons for ICU admission of medical patients were intoxications, pulmonary and infectious diseases. Mortality of study population was 9.0%, differing significantly between the groups according to the patients' profile (2.5% in elective surgical, 19.8% in emergency surgical and 31.4% in medical patients, p < 0.001, ANOVA).

### 2. Development and risk factors of GIF

GIF was detected in 252 patients during their ICU stay (Table 1). The incidence of GIF among patients with surgical and medical emergencies was significantly higher compared to elective cardiosurgical patients (18.2 % and 19.1 % vs. 5.7% respectively, p < 0.001).

**Figure 1:**
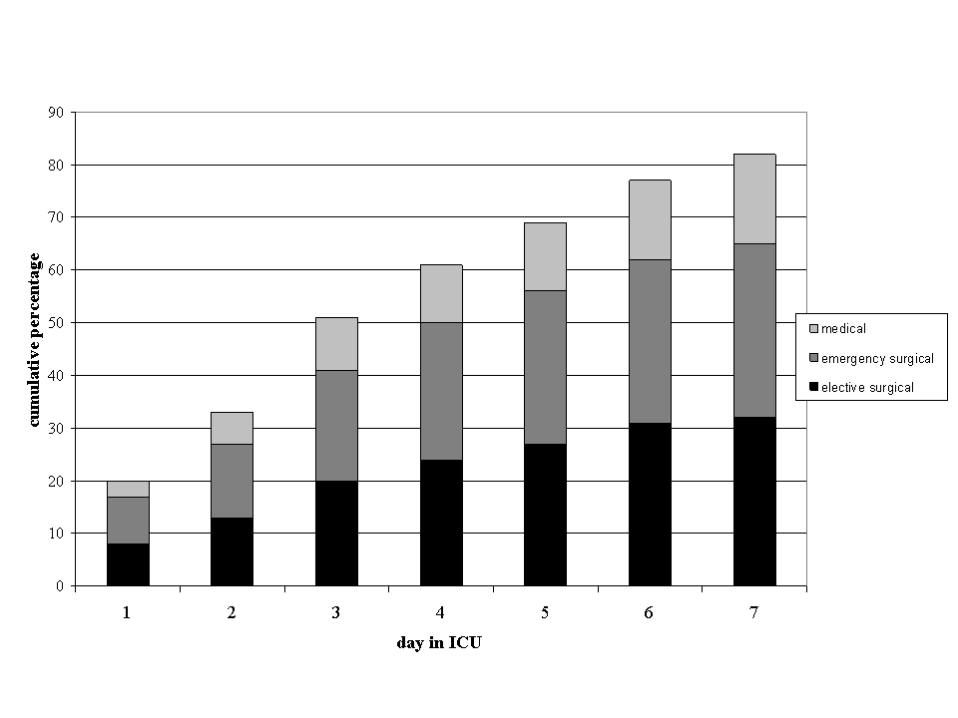
Cumulative incidence of gastrointestinal failure during the first week of stay in intensive care.

GIF developed late during ICU stay (Figure 1). On admission only 20% of all GIF cases were seen. 82% of GIF cases were clinically manifested by the end of the first week in ICU.

Twenty-three variables listed below, documented at the day of admission, were identified as highly predictive (p < 0.01) risk factors for GIF development: patients' age, medical profile, haematocrit, leucocyte count, platelet count, creatinine, urea, bilirubin, C-reactive protein, lactate, pO_2_/FiO_2_, mean arterial pressure, central venous pressure, APACHE II, SOFA; SAPS II, use of catecholamines, sedation, PEEP, hemodialysis, low-output syndrome, septic shock, and use of blood products.

In logistic regression analysis we identified the independent predictors for development of GIF resulting with the model including:

APACHE II (OR 1.05; 95%CI 1.02–1.09);

SOFA (OR 1.11; 95%CI 1.02–1.20);

patients' emergency profile (OR 3.09; 95%CI 2.11–4.52);

use of catecholamines (OR 4.16; 95%CI 2.82–6.15).

Even though the model allowed us to predict 92.3% from all of the cases correctly, only 19.8% of GIF (true-positive) cases could be correctly predicted. True-negative cases, i.e. nonGIF patients, in contrast, can be correctly predicted in 99.0% of cases.

### 3. ICU outcome and the risk of death associated with GIF

The development of GIF during the ICU treatment was associated with a significantly higher mortality, prolonged the ICU stay and duration of mechanical ventilation (Table 1). While the overall mortality of this mixed study population was 9.0 %, the mortality of patients with GIF was almost eight times of that of nonGIF patients (43.7% vs. 5.3%, p < 0.001).

Development of GIF during the ICU stay (or its presence on admission) increased the risk of death markedly in the overall study population (OR 13.94; 95%CI 10.18–18.92). In particular, the elective cardiosurgical patients had tremendously greater likelihood to die if they developed GIF during the ICU stay (OR 31.82; 95%CI 16.61–60.95). The risk of death was also significantly increased for surgical (OR 5.33; 95%CI 3.36–8.46) and medical emergency patients (OR 13.64; 95%CI 6.22–29.94).

In multiple logistic regression analysis APACHE II and SOFA scores at admission and development of GIF during ICU stay were identified as independent risk factors for death.

The models for prediction of ICU death are described in Table 2.

## Discussion

The present study demonstrates that GIF represents a relevant clinical predictor of mortality in intensive care patients. GIF significantly prolonged mechanical ventilation and ICU stay. This study suggests the need for further investigation and discussion in order to develop a proper clinical definition and an evaluation system for gastrointestinal failure.

GIF was observed less frequently in patients following elective cardiac surgery. Indeed, patients admitted for surgical or medical emergencies developed gastrointestinal problems much more frequently and often presented with these symptoms on admission. However, regardless of ICU location or patient profile, the analysis consistently revealed that development of GIF during patients' ICU treatment resulted in a significant increase in the duration of mechanical ventilation, length of stay and, most importantly, of ICU mortality. The overall mortality (GIF and nonGIF) in elective cardiosurgical patients was 2.5 %, while in patients without GIF it was very low – only 1.1%. By contrast, the influence of GIF development on mortality was tremendous: the risk of death showed a twenty-three-fold increase. This is in accordance with recent analysis of cardiac surgery patients demonstrating that development of postoperative organ failures (respiratory failure, acute renal failure) significantly prolongs the ICU stay and is associated with lower survival [12]. The importance of gastrointestinal function in cardiosurgical patients has been addressed in other recent studies. Hessel demonstrated, that gastrointestinal complications occur in about 2.5% of patients undergoing cardiac surgery and that the occurrence of these complications is associated with a high mortality [13]. Ishikawa et al. described similar results [14].

In our study, the risk of death was significantly increased also among emergency surgical and medical patients with GIF. One possible tool for the assessment of GI function, measurement of intraabdominal pressure, was not performed in our patients. In recent studies, intraabdominal hypertension has been associated with worse outcome in mixed critically ill patients [10]. Still, it needs to be clarified, whether the measurement of intra-abdominal pressure alone is sufficient for complex assessment of GI function [15].

The impact of GIF on ICU mortality and length of stay clearly points out the need for predictors for GIF using patient characteristics on the day of admission. Identifying the population at risk would clearly be beneficial, allowing the practitioner to apply necessary strategies to prevent the development of the syndrome. Even though we identified several highly predictive parameters and four independent predictors for GIF development, the attempts to develop an equation correctly predicting the manifestation of GIF were not successful. There are several explanations for this. One involves the categorisation of patients according to the presence or absence of GIF. It is obvious that organ failure is not an all-or-nothing phenomenon, but a progression of alterations from normal organ function to organ failure [16]. GIF is a syndrome with a variable onset during ICU treatment. In their original study introducing the MOF score, Goris et al. noticed that the mean onset of GI failure was on the 23^rd ^ICU day in severe trauma patients and on the 11^th ^day in patients with septic intra-abdominal complications following surgery [6]. GIF was defined as cholecystitis, stress ulcer, GI haemorrhage, necrotic enterocolitis or pancreatitis and/or spontaneous perforation of the gallbladder. In the present study, 80% of GIF patients were identifiable by the end of the 1^st ^week of stay in the ICU, while 20% developed GIF later. Length of stay in the ICU was significantly longer in GIF patients compared to non-GIF patients. All these results indicate that development of GIF may occur late in a patients' ICU stay. This could prove to be one of the main problems in GIF prediction based on patient data at admission. It is possible that not the variables at admission, but changes in these characteristics following the first day of treatment, could prove more reliable for prediction of GIF.

In the opinion of the authors, the absence of a consensus definition of GIF is a major limiting factor of research on the area. The absence of a clear definition of GIF and the retrospective design should also be considered as main limitations of present study. The symptoms used for the diagnosis of GIF were somewhat vaguely specified and that may interfere with the results. Further, the severity of the GI symptoms was not accounted – for example, food intolerance and gastrointestinal haemorrhage were weighted uniformly. While definitions and grading based on defined clinical signs and laboratory findings are available for most organ failures – for example, ARDS and ALI in respiratory failure, NYHA classes in heart failure, renal dysfunction and failure – a well-accepted definition of gastrointestinal failure is absent. It has been suggested that the assessment of gastrointestinal function, being subjective and hardly measurable, would have negligible impact on the scoring of organ functions for subsequent prediction of ICU performance [7]. In the present study development of GIF during ICU stay was identified as an independent risk factor for death. However, it should be considered that GIF was mostly not present at admission but developed during the entire ICU period, while the other risk factors in our model: APACHE II and SOFA; were documented on day 1. Also it should be considered that APACHE II was originally validated for hospital mortality [11] while ICU mortality was assessed in our study. These facts are probably diminishing the practical value of our model for prediction of death.

The profile of patients studied should also be carefully considered – most of the patients were elective cardiosurgical patients. In our study, low output syndrome was a highly predictive parameter and the use of catecholamines an independent risk factor for GIF. This fact is probably appointing GIF development being associated with global hypoperfusion in patients with compromised cardiovascular system. The deteriorated gastrointestinal barrier function after cardiopulmonary bypass may also contribute to the development of GIF in cardiac surgery patients [17]. Cardiovascular SOFA category was not assessed separately in our analysis due to missing respective data, but might be of value in further studies, especially in cardiosurgical patients.

## Conclusion

GIF should be considered a relevant clinical predictor of increased mortality and prolonged ICU stay. Several risk factors for the development of GIF could be identified. The most dramatic risk of death was observed in elective cardiac surgery patients. However, predicting GIF remains still very complicated. Further studies, defining the syndrome of gastrointestinal failure and investigating its impact on ICU patients are warranted.

## Abbreviations

ICU – intensive care unit

GIF – gastrointestinal failure

APACHE – Acute Physiology and Chronic Health Evaluation

SOFA – Sequential Organ Failure Assessment

MOF – multiple organ failure

ANOVA – Analysis of variance

OR – odds ratio

95%CI – 95% confidence interval

ARDS – adult respiratory distress syndrome

ALI – acute lung injury

NYHA – New York Heart Association

## Competing interests

The author(s) declare that they have no competing interests.

## Authors' contributions

AR participated in the design of the study and drafted the manuscript. PP and UR carried out the data collection. LT participated in the statistical analysis. JS participated in the design of the study and writing the manuscript. UR carried out the data collection in Berlin and participated in the statistical analysis. FK participated in the design of the study and the coordination between the different centres. CS participated in the design of the study, the statistical analysis and writing the manuscript. HK conceived the study, and participated in its design and coordination, and helped to draft the manuscript. All authors read and approved the final manuscript.

## Pre-publication history

The pre-publication history for this paper can be accessed here:



## Figures and Tables

**Table 1:** Incidence, characteristics and outcome of gastrointestinal failure (GIF) in intensive care patients. Data are presented as medians (with lower and upper quartiles) if not stated otherwise. P-values (Mann-Whitney U-test for continuous variables or chi square test for categorical variables) are calculated for the differences between GIF and nonGIF patients

	**GIF**	**NonGIF**	**Total**	**P-value (GIF vs. nonGIF)**
Number of patients (% from total patients)	252 (9.7%)	2336 (90.3%)	2588 (100.0%)	
Female/male ratio; patients	76/176	820/1516	896/1692	0.066
Age; years	67 (60–74)	64 (54–72)	64 (55–72)	<0.001
APACHE II score on admission; points	18 (14–26)	12 (9–16)	13 (9–17)	<0.001
SOFA score on admission; points	9 (6–11)	5 (4–7)	5 (4–7)	<0.001
Mechanically ventilated; patients (%)	226 (89.7%)	1930 (82.6%)	2156 (83.3%)	0.003
Mechanical ventilation; days	8.0 (3.0–19.0)	1.0 (1.0–1.0)	1.0 (1.0–2.0)	<0.001
ICU stay; days	10.0 (6.0–23.0)	2.0 (2.0–3.0)	2.0 (2.0–4.0)	<0.001
ICU mortality; patients (%)	110 (43.7%)	124 (5.3%)	234 (9,0%)	<0.001

**Table 2:** Logistic regression models for prediction of death in whole study population and in different groups according to patients' profile

	**Total**	**Elective surgical**	**Emergency surgical**	**Medical**
*Predictors*	*p-value* *OR (95% CI)*	*p-value* *OR (95% CI)*	*p-value* *OR (95% CI)*	*p-value * *OR(95% CI)*
APACHE II	0.020 1.03 (1.01–1.06)	0.031 1.06 (1.01–1.13)	0.730 1.01 (0.97–1.04)	0.259 1.03 (0.98–1.09)
SOFA	<0.001 1.35 (1.27–1.44)	0.013 1.20 (1.04–1.39)	<0.001 1.30 (1.20–1.41)	<0.001 1.36 (1.19–1.55)
GIF	<0.001 7.44 (5.21–10.62)	<0.001 15.42 (7.67–31.04)	<0.001 3.31 (1.97–5.55)	<0.001 7.43 (3.00–18.36)
*Pseudo R-square Nagelkerke's coeff.*	0.40	0.35	0.33	0.56
